# Glycosylphosphatidylinositol-Anchor Synthesis in Plants: A Glycobiology Perspective

**DOI:** 10.3389/fpls.2020.611188

**Published:** 2020-11-17

**Authors:** Gernot Beihammer, Daniel Maresch, Friedrich Altmann, Richard Strasser

**Affiliations:** ^1^Department of Applied Genetics and Cell Biology, Institute of Plant Biotechnology and Cell Biology, University of Natural Resources and Life Sciences, Vienna, Austria; ^2^Division of Biochemistry, Department of Chemistry, University of Natural Resources and Life Sciences, Vienna, Austria

**Keywords:** posttranslational modification, mannose, glycosyltransferase, glycosylation, endoplasmic reticulum, glycosylphosphatidylinositol

## Abstract

More than 200 diverse secretory proteins from *Arabidopsis thaliana* carry a glycosylphosphatidylinositol (GPI) lipid anchor covalently attached to their carboxyl-terminus. The GPI-anchor contains a lipid-linked glycan backbone that is preassembled in the endoplasmic reticulum (ER) of plants and subsequently transferred to distinct proteins, which provides them with specific features. The GPI-anchored proteins exit the ER and are transported through the Golgi apparatus to the plasma membrane. In the Golgi, the glycan moiety can be further modified by the specific attachment of sugar residues. While these biosynthetic steps are already quite well understood in mammals and yeast, comparatively little is known in plants. In this perspective, we discuss the current knowledge about the biosynthesis of the GPI-anchor glycan moiety in the light of recent findings for mammalian GPI-anchor glycan modifications.

## Introduction

**Figure 1:**
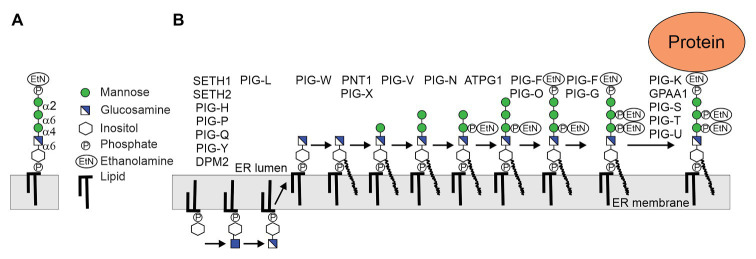
**(A)** Structure of the conserved glycosylphosphatidylinositol (GPI)-anchor backbone. **(B)** Biosynthesis of plant GPI precursor in the endoplasmic reticulum (ER). The biosynthesis is a stepwise process initiated at the cytoplasmic side of the ER by the GPI-GlcNAc-transferase (GPI-GnT) multiprotein complex (SETH1, SETH2, PIG-H, PIG-P, PIG-Q, PIG-Y, and DPM2). The GlcNAc is de-N-acetylated by PIG-L and GlcN-PI flips to the luminal side of the ER. GPI mannosyltransferases PNT1 (homolog of mammalian PIG-M), PIG-V and ATPG1 (homolog of mammalian PIG-B) attach the three mannose (Man) residues to the backbone, which is further modified by attachment of ethanolamine phosphate (EtNP). The assembled precursor is transferred en bloc by the GPI transamidase complex (PIG-K, GPAA1, PIG-S, PIG-T, and PIG-U) to proteins. In addition to the bridging EtNP, one or two additional EtNP may be transferred to mannose residues by specific GPI-EtNP transferases that are present in *Arabidopsis thaliana* (Ellis et al., 2010; Luschnig and Seifert, 2011).

The attachment of glycosylphosphatidylinositol (GPI) is a common posttranslational modification for anchoring of proteins to the outer surface of the plasma membrane in eukaryotes. The conserved GPI moiety is composed of ethanolamine phosphate (EtNP), a conserved core glycan and phosphatidylinositol. The core glycan consists of three mannoses (Man) and one glucosamine (GlcN) residue that are linked to EtNP and phosphatidylinositol forming the GPI backbone structure EtNP-6Manα1-2Manα1-6Manα1-4GlcNα1-6*myo*-inositol-phospholipid (Kinoshita and Fujita, 2016; Liu and Fujita, 2020; Figure 1). Proteins destined to be GPI-anchored are translocated into the lumen of the endoplasmic reticulum (ER), the GPI attachment signal peptide at the C-terminus is cleaved off and the preassembled GPI is transferred en bloc to the last amino acid of the C-terminus called the ω site. The transfer is mediated by the GPI transamidase, a multi-subunit complex comprising five proteins. The attachment of GPI results in anchoring of the protein to the outer leaflet of the lipid bilayer. The modification with a GPI-anchor confers specific properties on proteins, such as efficient ER exit, sorting to the plasma membrane and association with specific membrane microdomains (Sikorska et al., 2016). In *Arabidopsis thaliana*, multiple protein families have been predicted by bioinformatic analysis to carry a GPI-anchor (Borner et al., 2002; Eisenhaber et al., 2003), and approximately 200 GPI-anchored proteins have been identified by different proteomics approaches (Borner et al., 2003; Elortza et al., 2006; Takahashi et al., 2016). Proteins that carry a GPI-anchor include the multi-copper oxidase-related protein SKU5 (Sedbrook et al., 2002), COBRA family proteins (Schindelman et al., 2001; Roudier et al., 2005; Brady et al., 2007), lipid-transfer proteins (LTPGs; Debono et al., 2009), and arabinogalactan proteins (AGPs; Oxley and Bacic, 1999; Shi et al., 2003; Xue et al., 2017). For a more comprehensive list of potential GPI-anchored proteins and phenotypes associated with mutants see recent reviews (Yeats et al., 2018; Zhou, 2019). Here, we focus on the biosynthesis of the GPI-anchor core glycan moiety and potential side chain modifications.

## GPI-Anchor Glycan Biosynthesis in Mammals and Yeast

In mammalian cells, at least 150 proteins at the plasma membrane are attached to the cell surface by GPI-anchors and GPI-anchoring is essential for many biological processes including embryogenesis, fertilization, or the immune system (Kinoshita and Fujita, 2016). In *Saccharomyces cerevisiae*, more than 60 GPI-anchored proteins have been identified and GPI biosynthesis is required for the growth of yeast (Leidich et al., 1994). During biosynthesis and after attachment to proteins, the structures of lipid and glycan moieties from GPI-anchors are remodeled in the ER and in the Golgi apparatus.

Glycosylphosphatidylinositol biosynthesis is initiated at the cytosolic side of the ER by the transfer of GlcNAc from the nucleotide sugar UDP-GlcNAc to inositol to generate GlcNAc-PI (Figure 1). This step is catalyzed by the GPI-GlcNAc-transferase (GPI-GnT), a complex consisting of seven protein subunits in mammalian cells. GlcNAc-PI is de-N-acetylated to GlcN-PI by the deacetylase PIG-L, and GlcN-PI is flipped to the luminal side by an unknown process. In the ER lumen, GlcN-PI is acylated by the acyltransferase PIG-W and the lipid moiety is remodeled to generate GlcN-(acyl)PI. In the next step, the GPI α1,4-mannosyltransferase PIG-M and the GPI α1,6-mannosyltransferases PIG-V catalyze the sequential transfer of two Man residues to GlcN-(acyl)PI (Maeda et al., 2001; Kang et al., 2005). PIG-B, another GPI α1,2-mannosyltransferase, transfers the third mannose to generate Manα1-2Manα1-6Manα1-4GlcN-(acyl)PI (Takahashi et al., 1996). A GPI-EtNP-transferase transfers the so-called bridging EtNP that connects the protein and the glycan to the third mannose to generate EtNP-6Manα1-2Manα1-6Manα1-4GlcN-(acyl)PI (Kinoshita and Fujita, 2016). Two additional side-branch EtNPs are added to the first and second mannose residues. While the EtNP transfer to the first mannose takes place after the transfer of the second mannose, the modification of the second mannose with EtNP likely happens after the attachment of the bridging EtNP. The resulting structure is competent for transfer to proteins, but can be further modified by the attachment of a fourth mannose residue catalyzed by the GPI α1,2-mannosyltransferase PIG-Z. Like the ER-resident mannosyltransferases (ALG3, ALG9, and ALG11) involved in the assembly of the oligosaccharide precursor for N-glycosylation, all four GPI mannosyltransferases are multiple transmembrane proteins and use dolichol-phosphate-mannose (Dol-P-Man) as donor substrate. PIG-B and PIG-Z are like ALG9 and ALG12 members of CAZy family GT22. PIG-V which is distantly related to STT3, the catalytic subunit of the oligosaccharyltransferase complex, belongs to family GT76 (Kang et al., 2005). PIG-M is distantly related to ALG3 (GT58) and belongs to GT50 (Oriol et al., 2002). All these enzymes use dolichol-linked donor substrates and have 11–14 membrane spanning helices and conserved residues in luminal loops (Albuquerque-Wendt et al., 2019).

In yeast, an α1,2-linked mannose is attached to the third mannose and this modification is an essential biosynthetic step in the assembly of yeast GPIs (Grimme et al., 2001). This mannosylation step takes place in the ER and precedes the attachment of the bridging EtNP. In mammals, some GPI-anchored proteins have also a fourth mannose residue in the same position that is transferred by PIG-Z (Taron et al., 2004; Hirata et al., 2018). In addition, the first mannose residue of mammalian GPI-anchors is often modified with N-acetylgalactosamine (GalNAc). In contrast to the previously mentioned glycosylation reactions, this step takes place in the Golgi apparatus and is catalyzed by the GPI GalNAc-transferase PGAP4 (Hirata et al., 2018). This enzyme uses UDP-GalNAc as donor substrate. The GalNAc side chain may be further elongated by incorporation of β1,3-linked galactose and α2,3-linked sialic acid. The Golgi-resident galactosyltransferase B3GALT4 catalyzes the transfer of a galactose from UDP-galactose to the side chain GalNAc residue (Wang et al., 2020). The GPI sialyltransferase was not identified yet, but recently it was found that in prion protein the sialic acid N-acetylneuraminic acid (Neu5Ac) is present in α2,3-linkage (Kobayashi et al., 2020).

## GPI-Anchor Core Glycan Biosynthesis in Plants

In contrast to mammals and yeast, our knowledge about the different biosynthetic steps involved in the assembly of the GPI core glycan and possible side chain formations is limited (Yeats et al., 2018). Based on sequence comparison, *Arabidopsis* SETH1 and SETH2 have been identified as homologs of subunits PIG-C and PIG-A of the GPI-GnT complex (Lalanne et al., 2004). Disruption of SETH1 or SETH2 affects pollen germination and tube growth suggesting a role of GPI-anchored proteins in pollen function. PEANUT1 (PNT1) is the *Arabidopsis* homolog of PIG-M involved in the first mannosylation step (Gillmor et al., 2005; Figure 1). The *pnt1* mutant is embryo lethal, displays defects in cell wall biosynthesis and GPI-anchored proteins like SKU5 or COBRA are absent or strongly reduced in *pnt1* embryos or callus. Mammalian PIG-M forms a complex with PIG-X, which stabilizes the catalytic subunit PIG-M (Ashida et al., 2005). The *Arabidopsis* PIG-X homolog (At5g46850) has not been characterized but likely has a similar function. An *Arabidopsis* PIG-X knockout could display a less severe phenotype than *pnt1* because PIG-M expression is not completely abolished in mammalian cells lacking PIG-X (Ashida et al., 2005). *Arabidopsis* lines lacking PIG-V, the GPI mannosyltransferase catalyzing the transfer of the second mannose, have not been described and PIG-V has not been biochemically characterized. Like *pnt1*, a complete PIG-V knockout will block the biosynthesis of the GPI backbone and is thus likely embryo lethal. *Arabidopsis* ABNORMAL POLLEN TUBE GUIDANCE1 (APTG1) can functionally replace the yeast PIG-B homolog that transfers the third mannose to the GPI precursor (Dai et al., 2014). In line with the essential function in plants, the *aptg1* mutant showed embryo lethality. APTG1 is an integral membrane protein located in the ER and plants with disrupted APTG1 expression display mislocalization of GPI-anchored proteins. Together these studies provide a clear and consistent insight into the enzymes involved in the core glycan biosynthesis and their biological function in plants.

## Side Chain Modifications in Plants

The complete chemical structure of a mammalian GPI-anchor was published in 1988 (Homans et al., 1988). In plants, only one study reported the structure of a GPI-anchored protein (Oxley and Bacic, 1999). The GPI-anchor of an AGP isolated from *Pyrus communis* suspension cells has a glycan core that is identical to the one from mammals and yeast. Instead of a GalNAc at the same position, the first mannose of the core glycan was partially modified with a β-linked galactose.

**Figure 2:**
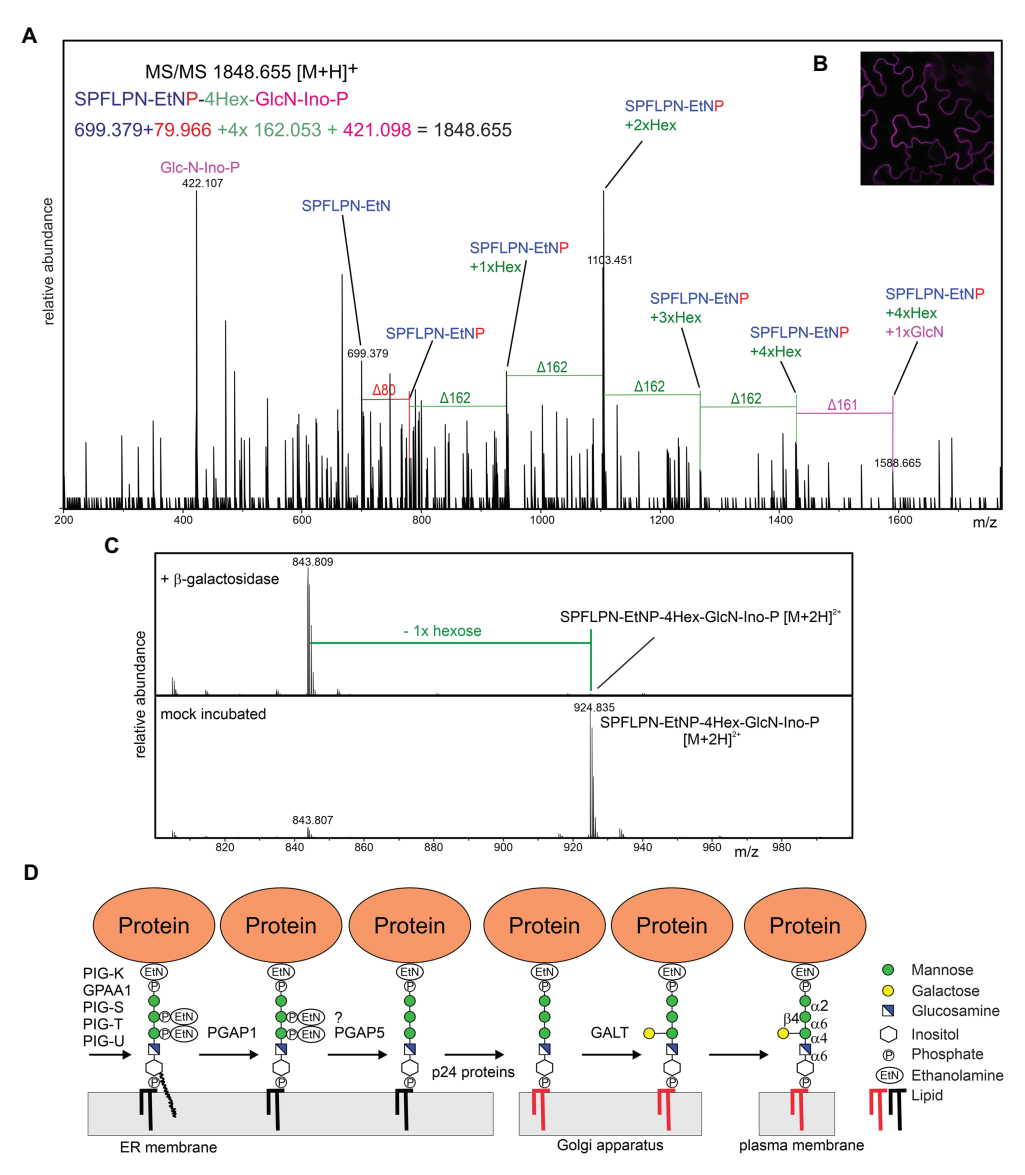
**(A)** LC-ESI-MS/MS analysis of the C-terminal peptide from RFP-COB1. RFP-COB1 was expressed in *A. thaliana* seedlings, captured by binding to RFP-Trap resin and subjected to PI-PLC and trypsin digestion. **(B)** RFP-COB1 is found at the plasma membrane. RFP-COB1 was transiently expressed in *Nicotiana benthamiana* leaf epidermal cells and analyzed by confocal microscopy. **(C)** LC-ESI-MS analysis of β-galactosidase digested GPI-anchor derived from *A. thaliana* expressed RFP-COB1. **(D)** The transferred GPI backbone is further modified by different enzymes, including inositol deacylation, the remodeling of the lipid portion (depicted by the change in color from black to red in the illustration) and the EtNP removal, which is likely required for recognition by p24 cargo receptor proteins and efficient ER exit. In the proposed model, the plant-specific side chain modification is transferred in the Golgi by an unknown β-galactosyltransferase (GALT).

To see if the presence of the β-linked galactose is common in plants, we transiently expressed RFP fused to the C-terminal GPI attachment signal peptide from *Arabidopsis* COBRA transiently in *Nicotiana benthamiana* leaves or in transgenic *Arabidopsis* (Strasser et al., 2005), purified the GPI-anchored RFP-COB1 reporter protein and subjected the PI-PLC released C-terminal tryptic peptide to LC-ESI-MS/MS analysis (Kolarich and Altmann, 2000) (Figure 2). RFP-COB1 was present in the plasma membrane and MS-spectra of the terminal peptide from the two different plant species were obtained. The MS-analysis revealed masses in both species corresponding to the presence of the GPI core backbone without any additional side chain EtNP modifications. The presence of the respective EtNP transferases in the *Arabidopsis* genome (Ellis et al., 2010; Luschnig and Seifert, 2011) suggests that this modification is removed during the remodeling of the GPI-anchor rather than being absent in plants. In mammalian cells, the side chain EtNP is removed by the phosphoesterase PGAP5 (Fujita et al., 2009). While yeast contain two PGAP5 homologs (CDC1 and TED1), there is one (At1g53710) so far uncharacterized PGAP5 homolog in *Arabidopsis*. Structures composed of four hexoses were detected in RFP-COB1 from *A. thaliana* (Figure 2) and *N. benthamiana* (Supplementary Figure S1), which is consistent with the three sequential mannoses in the core glycan. The fourth hexose is a side chain modification that is likely the previously described β-linked galactose. Alternatively, the hexose could be a mannose residue α-linked to the third mannose that has been described in yeast and mammals. When, we digested the peptide from RFP-COB1 with a β-galactosidase, a single hexose (mass ∆162) was quantitatively removed from the *Arabidopsis* derived peptide (Figure 2). For the peptide from *N. benthamiana*, we performed an additional α-mannosidase digestion because the β-galactosidase treatment did not fully remove the hexose (Supplementary Figure S2). The α-mannosidase treatment did not alter the glycan composition of the GPI-anchor suggesting that the fourth hexose is a galactose. It seems likely that the galactose is bound in β1,4-linkage because the galactosidase used for the digestion exhibits high specificity for this type of linkage (Zeleny et al., 1997). Closer investigation of the MS/MS-spectra of RFP-COB1 subjected to β-galactosidase treatment revealed that the mass corresponding to GlcN-PI+2xHex was absent when compared to the mock incubated control, indicating that the galactose is bound to the GlcN-linked mannose (Supplementary Figure S3). These findings are consistent with the structure from AGP isolated from pear cells (Oxley and Bacic, 1999) indicating that attachment of a single galactose in β-linkage is a common side chain formation of the GPI core glycan in plants.

## Discussion

The detection of a single hexose as GPI side chain modification raises several questions: which glycosyltransferase catalyzes this step, which donor substrate is used in the reaction and in which subcellular compartment is the side chain modified. The glycosyltransferase family CAZy GT22 (α1,2-mannosyltransferases) contains only three *Arabidopsis* proteins. APTG1, the only homolog of PIG-B and the two enzymes (ALG12 and ALG9) involved in the assembly of the lipid-linked oligosaccharide precursor for N-glycosylation. The lack of a PIG-Z homolog in plants (Ellis et al., 2010; Luschnig and Seifert, 2011) is consistent with the absence of a fourth mannose residue attached to the GPI glycan core in the ER unless APTG1 transfers a second mannose residue as it has been suggested for PIG-B (Wang et al., 2020). Many ER-resident glycosyltransferases are integral membrane proteins that use dolichol-phosphate-linked sugars as donor substrate. A Dol-P-galactose has not been described and ER-resident multiple transmembrane domain-containing galactosyltransferases are not known. Therefore, we suggest that the side chain modification takes place in the Golgi apparatus of plants and involves an unknown β-galactosyltransferase that uses UDP-galactose as donor substrate. Golgi glycosyltransferases are typically type II membrane proteins with a single transmembrane domain and a short N-terminal cytoplasmic region (Schoberer and Strasser, 2011). The recently identified GPI-GalNAc-transferase PGAP4 has a different structure with two additional tandem transmembrane domains (Hirata et al., 2018). This peculiar structure likely facilitates the interaction with the membrane-anchored substrate. While there is no PGAP4 homolog present in the *Arabidopsis* proteome, it is possible that the unknown plant GPI-galactosyltransferase has a similar membrane topology. On the other hand, B3GALT4 that transfers a galactose to the GalNAc has a common type II membrane topology. B3GALT4 is distantly related to *Arabidopsis* hydroxyproline O-galactosyltransferases that are members of the GT31 family (Basu et al., 2015). Plants have a large family of GT31 galactosyltransferases with still poorly characterized function (Showalter and Basu, 2016) that are involved in different pathways, including N-glycan processing (Strasser et al., 2007). One of those galactosyltransferases from GT31 could be responsible for the side chain formation of GPI core glycan.

What is the function of the GPI side chain modification? Like for many glycan modifications such as Lewis A-type structures, the function of the attached GPI side chain galactose is currently unknown (Strasser, 2016). In mammalian brain, galactosylated and sialylated GPI-anchors are more abundant than in other tissues. However, not all GPI-anchored proteins are modified to the same extent with some proteins having only GalNAc instead of additional galactose and sialic acid modifications (Kobayashi et al., 2020). Sialylation of the human prion protein side chain may contribute to the pathology of prion disease (Bate et al., 2016). The specific function of side chain glycosylation in mammals and the conserved nature of the identified core glycan modification in plants suggests that the β-linked galactose has a biological role that needs to be unraveled in future studies.

## Data Availability Statement

The original contributions presented in the study are included in the article/Supplementary Material, further inquiries can be directed to the corresponding author.

## Author Contributions

GB and RS designed the experiments and wrote the paper. GB and DM conducted the experiments. GB, DM, FA, and RS analyzed the results. All authors listed have made a substantial, direct and intellectual contribution to the work, and approved it for publication.

### Conflict of Interest

The authors declare that the research was conducted in the absence of any commercial or financial relationships that could be construed as a potential conflict of interest.

The reviewer VK declared a past co-authorship with several of the authors FA and RS to the handling editor.
