# Supplementary material for: Glycosylphosphatidylinositol-Anchor Synthesis in Plants: A Glycobiology Perspective
Source: Front Plant Sci. 2020 Nov 17;11:611188. doi: 10.3389/fpls.2020.611188 (PMC7704450; doi:10.3389/fpls.2020.611188)
Supplement: Supplementary file 1 [file Data_Sheet_1.pdf]

GPI-anchor synthesis in plants – a glycobiology perspective

Gernot Beihammer, Daniel Maresch, Friedrich Altmann, Richard Strasser

Supplementary Material

Contents

Figure S1. **LC-ESI-MS/MS analysis of the C-terminal peptide from *N. benthamiana* RFP-COB1.**

Figure S2. LC-ESI-MS analysis of α-mannosidase or β-galactosidase digested GPI-anchor derived from *N. benthamiana* expressed RFP-COB1.

Figure S3. LC-ESI-MS/MS analysis of β-galactosidase digested and mock-incubated GPI-anchor derived from RFP-COB1 expressed in *A. thaliana* seedlings.

Supplementary Methods

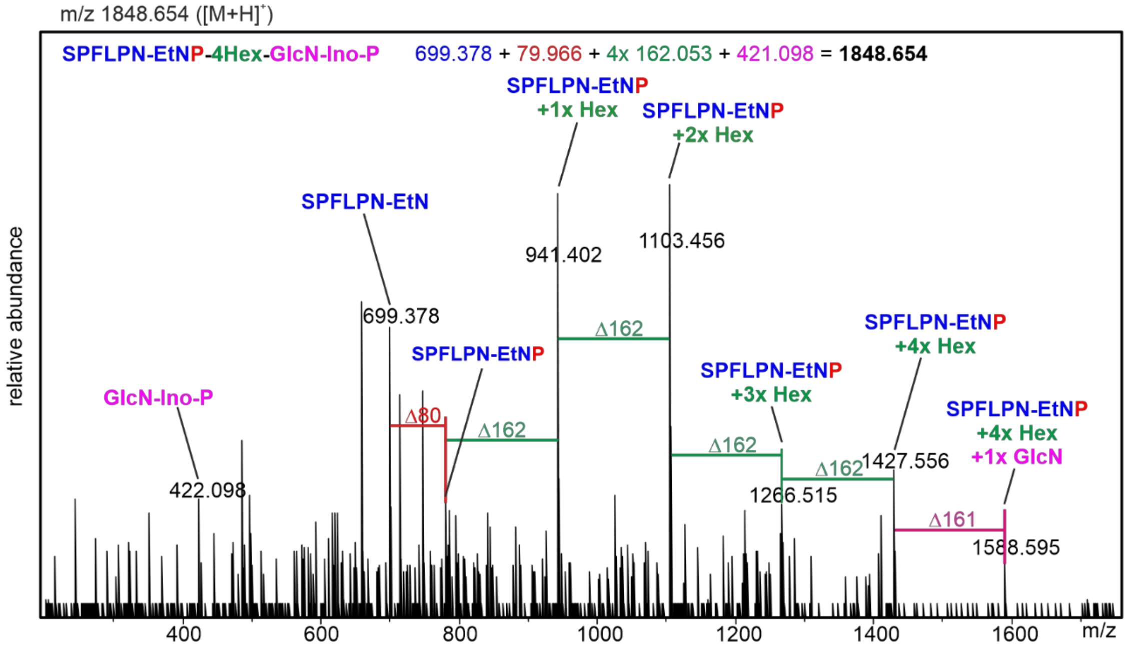

Figure S1. LC-ESI-MS/MS analysis of the C-terminal peptide from *N. benthamiana* RFP-COB1. RFP-COB1 was transiently expressed by agroinfiltration of leaves, captured by binding to RFP-Trap resin and subjected to PI-PLC and trypsin digestion. A representative analysis from two biological replicates is shown.

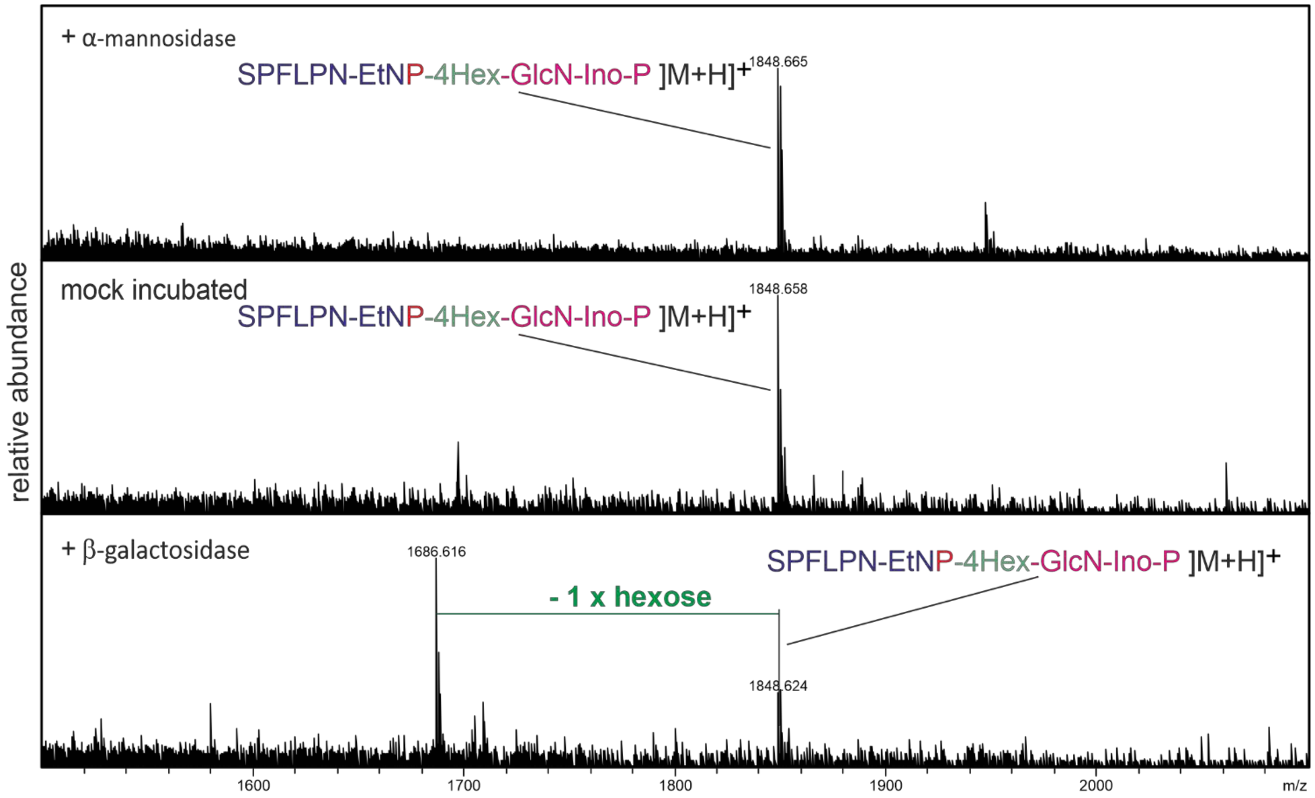

Figure S2. LC-ESI-MS analysis of α-mannosidase or β-galactosidase digested GPI-anchor derived from *N. benthamiana* expressed RFP-COB1. The β-galactosidase digestion was carried out two times with identical results. A representative analysis is shown.

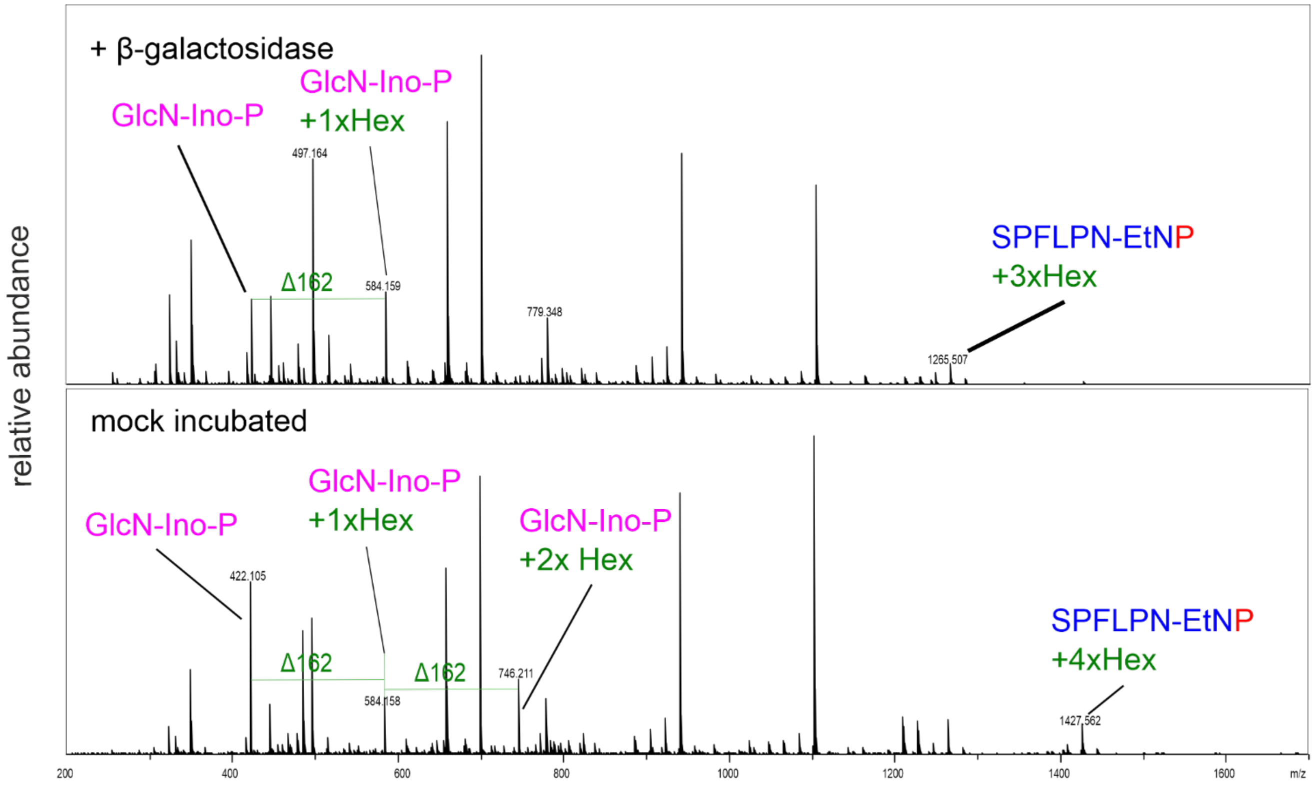

Figure S3. LC-ESI-MS/MS analysis of β-galactosidase digested and mock-incubated GPI-anchor derived from RFP-COB1 expressed in *A. thaliana* seedlings. A representative analysis from two technical replicates is shown.

Supplementary Methods

The C-terminal peptide of COBRA_ARATH was amplified from genomic DNA of *A. thaliana* using primers cob1_5F (5'-TATAGGATCCCCTTTTCTTCCCAACGGTGGTTCC-3')/cob1_6R (5'-TATACTCGAGTTAGGCAGAGAAGAAGAAAAAGAC-3') and cloned into a modified version of the pPT2-vector (Strasser *et al.* 2005) containing the Arabidopsis ubiquitin10-promotor and N-terminal mRFP. Reporter proteins were either purified from leaves of *N. benthamiana* following agroinfiltration or from 14-day old seedings of stably transformed *A. thaliana*. Plant samples were snap frozen, grinded and the membrane fraction collected by centrifugation in 100 mM Tris, 25 %(w/v) sucrose and 5 % (v/v) glycerol buffer (pH 7.4) at 65000 g for 1 h at 4 °C. After washing in 100 mM Tris, 150 mM NaCl and 0.5 mM EDTA buffer (pH 7.4), the membrane pellet was resuspended in 20 mM Tris, 10 mM NaCl, 0.1 mM $CaCl_2$, 1% (v/v) Phosphoinositide Phospholipase C (PIPLC, P6466, Thermo Fisher Scientific) (pH7.4) and incubated for 15 min. After cleavage of lipids from RFP-GPI, the protein was purified using RFP trap (Chromotek), separated by SDS-PAGE, excised from the gel, reduced, S-carbamidomethylated and subjected to tryptic digest as described previously (Kolarich and Altmann 2000). The resulting GPI-bearing peptide fragment was analyzed via LC-ESI-MS/MS using a Bruker Maxis 4G Q-TOF instrument. For enzymatic deglycosylation of the GPI anchor, either β-galactosidase from *Aspergillus oryzae* (Zeleny *et al.* 1997) or α-mannosidase from *Canavalia ensiformis* (M7257, Sigma-Aldrich) was used. RFP-GPI was purified once from *A. thaliana* and two times from *N. benthamiana*.

References

Kolarich, D. and Altmann, F. (2000). N-Glycan analysis by matrix-assisted laser desorption/ionization mass spectrometry of electrophoretically separated nonmammalian proteins: application to peanut allergen Ara h 1 and olive pollen allergen Ole e 1. Anal Biochem. 285, 64-75.

Strasser, R., Stadlmann, J., Svoboda, B., Altmann, F., Glössl, J. and Mach, L. (2005). Molecular basis of N-acetylglucosaminyltransferase I deficiency in *Arabidopsis thaliana* plants lacking complex N-glycans. Biochem J. 387, 385-91.

Zeleny, R., Altmann, F. and Praznik, W. (1997). A capillary electrophoretic study on the specificity of beta-galactosidases from *Aspergillus oryzae*, *Escherichia coli*, *Streptococcus pneumoniae*, and *Canavalia ensiformis* (jack bean). Anal Biochem. 246, 96-101.
